# Lung Cancer Screening—Trends and Current Studies

**DOI:** 10.3390/cancers16152691

**Published:** 2024-07-29

**Authors:** Aleksandra Czerw, Andrzej Deptała, Olga Partyka, Monika Pajewska, Ewa Wiśniewska, Katarzyna Sygit, Sławomir Wysocki, Elżbieta Cipora, Magdalena Konieczny, Tomasz Banaś, Krzysztof Małecki, Elżbieta Grochans, Szymon Grochans, Anna M. Cybulska, Daria Schneider-Matyka, Ewa Bandurska, Weronika Ciećko, Jarosław Drobnik, Piotr Pobrotyn, Urszula Grata-Borkowska, Joanna Furtak-Pobrotyn, Aleksandra Sierocka, Michał Marczak, Remigiusz Kozlowski

**Affiliations:** 1 Department of Health Economics and Medical Law, Medical University of Warsaw, 01-445 Warsaw, Poland; 2 Department of Economic and System Analyses, National Institute of Public Health NIH-National Research Institute, 00-791 Warsaw, Poland; 3 Department of Oncology Propaedeutics, Medical University of Warsaw, 01-445 Warsaw, Poland; 4 Faculty of Health Sciences, Calisia University, 62-800 Kalisz, Poland; 5 Provincial Specialised Healthcare Complex for Lung Diseases and Tuberculosis in Wolica, 62-872 Wolica, Poland; 6 Medical Institute, Jan Grodek State University in Sanok, 38-500 Sanok, Poland; 7 Department of Radiotherapy, Maria Sklodowska-Curie Institute-Oncology Center, 31-115 Cracow, Poland; 8 Department of Radiotherapy for Children and Adults, University Children’s Hospital of Cracow, 30-663 Cracow, Poland; 9 Department of Nursing, Faculty of Health Sciences, Pomeranian Medical University in Szczecin, 71-210 Szczecin, Poland; 10 Department of Pediatric and Oncological Surgery, Urology and Hand Surgery, Faculty of Medicine and Dentistry, Pomeranian Medical University in Szczecin, 71-252 Szczecin, Poland; 11 Center for Competence Development, Integrated Care and e-Health, Medical University of Gdansk, 80-204 Gdansk, Poland; 12 Department of Family Medicine, Faculty of Medicine, Wroclaw Medical University, 51-141 Wroclaw, Poland; 13 Remedial Specialistic Clinic, Pulsantis Sp. z o.o, 53-238 Wroclaw, Poland; 14 Citodent Dental Center Furtak-Pobrotyn & Company Limited Partnership, 05-220 Olawa, Poland; 15 Department of Management and Logistics in Healthcare, Medical University of Lodz, 90-131 Lodz, Poland; 16 Collegium of Management, WSB University in Warsaw, 03-204 Warsaw, Poland

**Keywords:** lung cancer, LM, screening, clinical trials

## Abstract

**Simple Summary:**

Lung cancer is one of the leading causes of cancer deaths around the world. The main risk factor associated with development of this disease is smoking, with about 80% of lung cancer deaths being related to smoking. However, non-smokers can also develop lung cancer due to an exposure to other harmful substances such as asbestos, radon and other carcinogens. The aim of our study is to present the trends and most recent studies aimed at lung cancer screening. Further research aimed at screening and smoking cessation interventions is recommended.

**Abstract:**

Lung cancer is the leading cause of death among all the oncological diseases worldwide. This applies to both women and men; however, the incidence and mortality among women is on the rise. In 2020, lung cancer was responsible for 1.8 million deaths (18%). More than 90% of lung cancer cases and 77.1% of lung cancer deaths occur in countries with high and very high HDI (human development index) values. The aim of our study is to the present trends and most recent studies aimed at lung cancer screening. In the face of the persistently high mortality rate, conducting research aimed at extending already-implemented diagnostic algorithms and behavioural interventions focused on smoking cessation is recommended.

## 1. Introduction

Based on the data from the World Health Organization, lung cancer is the number one cause of death among all the oncological diseases worldwide. This applies to both women and men. In 2020, lung cancer was responsible for 1.8 million deaths (18%). It was estimated that in 2020, 2.2 million new cases of lung cancer were diagnosed. The age-standardised incidence rate ranged from 5.9/100,000 in Mexico to 36.8/100,000 in Denmark. The age-standardised mortality rate ranged from 4.9/100,000 in Mexico to 32.8/100,000 in Poland. More than 90% of lung cancer cases and 77.1% of lung cancer deaths occur in countries with high and very high HDI (human development index) values [1,2].

The main risk factor for lung cancer is smoking, which is responsible for 85% of cases. Smoking behaviours at diagnosis may affect the prognosis [3]. The results of the analysis regarding lung cancer death rates in the period 1991–2018 showed that participants who had never smoked had an 86% lower risk of lung cancer death [4]. However, smoking-attributable lung cancer death rates decreased by 2.7% per year while smoking-unrelated lung cancer death rates declined 1.8% per year.

Other risk factors include passive smoking, environmental factors related to professional activities such as the presence of asbestos and radon, air pollution, a family history of cancer and chronic lung diseases. Seven epidemiological models have been developed, which, based on information about existing risk factors, allow for the estimation of the probability of developing lung cancer [5]. These models use gender, age, ethnicity, BMI, education, smoking data, family history of cancer and exposure to environmental risk factors as predictors. The existence of hereditary lung cancer has not yet been convincingly proven; however, several genome-wide association studies (GWAS) have been published not only identifying the chromosome locus 15q25.1 as a region that increases the risk of falling ill, but as a region also positively correlated with smoking and stronger nicotine addiction in Caucasians, African Americans and Asians [6,7]. The value of these findings that could be used in the design of screening methods remains unexplored. Also, smoking-related genes like PRR11 and PRR11 co-expressed genes were analysed as potential predictors of lung cancer and for the potential efficacy of immune checkpoint therapy [8].

Primary prevention is based fundamentally on quitting smoking, promoting the establishment of tobacco-free zones, limiting the availability of tobacco and activities leading to a reduction in air pollution.

The most common symptoms of lung cancer are chronic cough, recurrent lung and bronchial infections, chest pain, shortness of breath, expectoration of sputum with blood, fatigue and weight loss. Early symptoms of lung cancer are usually mild, which delays the correct diagnosis.

The U.S. Preventive Services Task Force (USPSTF) emphasises that smoking and advanced age are the main risk factors for lung cancer [9]. Patients aged 50–80 who have smoked for at least 20 years or used to smoke for at least 20 years and have quit smoking in the last 15 years or less constitute a particular risk group. The criteria used can therefore be expressed as ≥20 pack years for the group of people aged 50–80 who have quit smoking in the last 15 years or are still smoking. In this group of people, it is recommended to perform screening based on low-dose chest computed tomography (LDCT) every year. In a study conducted on 53,454 patients at risk over five consecutive years [10] and in a study including data obtained from 13,195 patients at risk [11], it was confirmed that screening using LDCT reduces mortality as a result of developing lung cancer by 20% compared to screening based on chest X-ray and by 24% compared to the control group in which no screening was performed. Tests such as respiratory cytology, chest X-ray or tumour marker tests are indicated as less effective in screening than computed tomography [12,13]. However, computed tomography is not a 100% effective method [14]. Based on the results obtained, it is not possible to completely exclude lung cancer, even when using the recommended method of annual screening. It is therefore important to further improve both the methods of identifying patients at risk and the screening methods used.

## 2. Materials and Methods

The aim of the study is to present current trends in clinical trials for lung cancer screening.

The basis for the analysis was information collected from the largest global registry of clinical trials [15]. This registry is maintained by the US National Institute of Health and the National Library of Medicine. ClinicalTrial.Gov is the largest database containing information on over 488,000 clinical trials conducted in over 220 countries around the world. Due to the topics discussed, only studies relating to lung cancer were included in the analysis.

**Figure 1:**
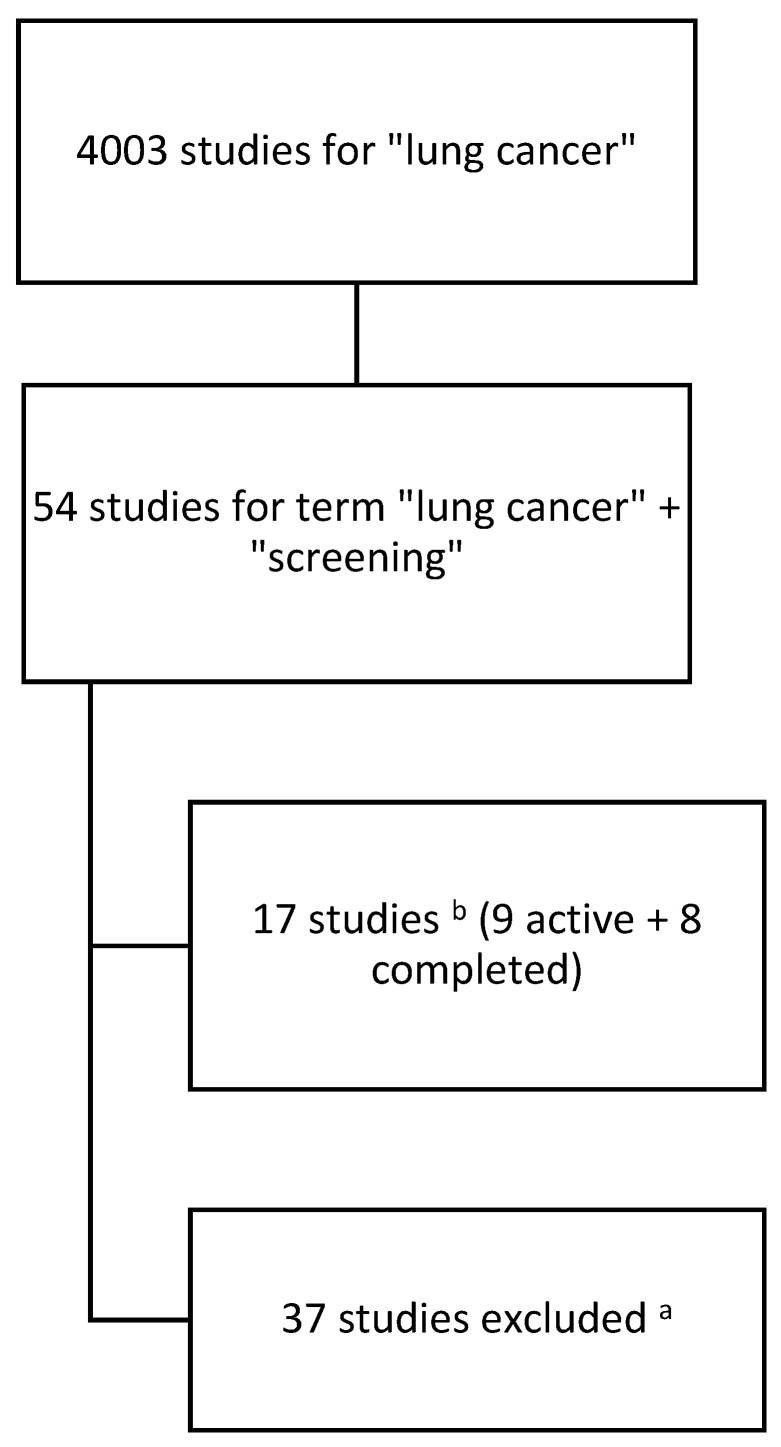
Process tree of searching and including studies into the analysis. a—studies with status: terminated, not yet recruiting, recruiting; b—in period of time 1 January 2019–31 March 2024.

The analysis included interventional and observational studies of which the main goal was the early detection of lung cancer. In order to present the current trend in research, the search period was narrowed to 2019–2024. Studies that had a completed and active status by 31 March 2024 have been taken into account in the analysis (see Figure 1).

Below is a process tree for including studies in the analysis:

## 3. Results

The data search identified a total of 17 studies, of which 9 were active and 8 were completed.

Table 1 presents the results of the analysis of collected materials regarding research in the field of the early detection of lung cancer during the period under study.

**Figure 2:**
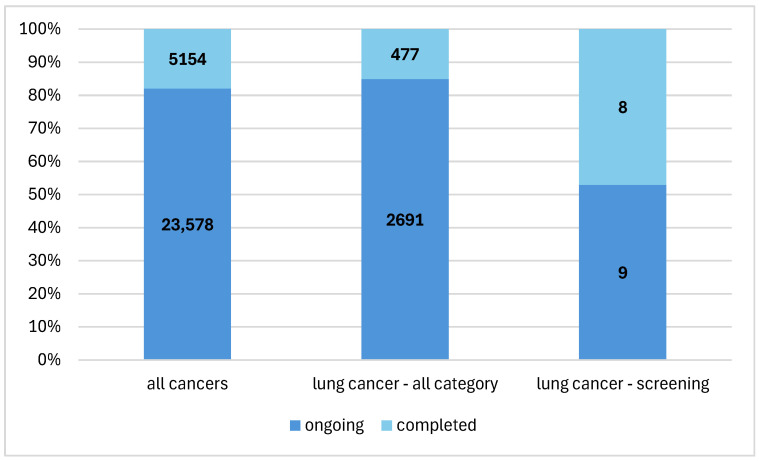
Percentage of research on early detection of lung cancer in the structure of cancer research.

In the entire database, there were 28,732 cancer studies registered in the years 2019–2024. Of this number, lung cancer studies collectively accounted for 11.0% (*n* = 3168) (Figure 2). This group also included research on therapeutic procedures in diagnosed patients, research on new drugs and research on patients in remission. Studies on the early detection of lung cancer accounted for 0.3% (*n* = 17) of all studies on lung cancer.

**Figure 3:**
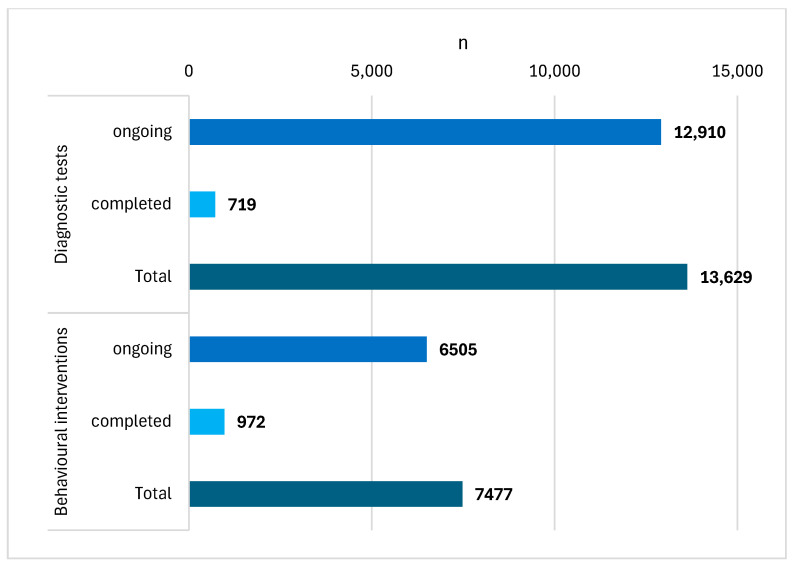
The number of participants combined was higher in the studies focused on diagnostic tests than in the studies focused on behavioural interventions.

Of the seventeen studies, four were started in 2019, three in 2020, another four in 2021, three in 2022 and three in 2023. One study applied to women only; in the remaining 16 studies, gender was not an inclusion criterion. Thirteen studies were interventional studies; the remaining four studies were observational studies. Sixteen studies focused only on adults. The sample sizes ranged from 8 to 12,000 study participants. Seven studies focused on behavioural interventions. Six studies focused on diagnostic tests. However, the number of participants combined was higher in the ongoing studies focused on diagnostic tests than in the ongoing studies focused on behavioural interventions (Figure 3).

The subject of the study conducted between 2022 and 2023 on 659 people (1) was to analyse the sensitivity and specificity of PCR-based tests in the diagnosis of lung cancer in relation to the results of computed tomography. In a study commenced in 2023 on a sample of 650 patients (2), work is underway to train an artificial intelligence model that is intended to estimate the risk of lung cancer based on the results of computed tomography and the results of markers in blood tests in relation to microRNA and C-reactive protein. In a study commenced in 2022 on a target sample of 100 patients (3), the possibility of using liquid DNA biopsy as a test preceding the decision to perform computed tomography has been verified. An IT tool is also being developed to analyse patients’ medical records in terms of meeting the inclusion criteria for screening using LDCT (4) and to provide information on the criteria and recommendations for screening to the doctor and the patient in electronic form. The next study (5) concerns the possibility of using chromatograms to analyse exhaled air in a group of patients diagnosed with non-small cell lung cancer (NSCLC) and in the control group in order to develop a screening method. Correlates of lung diseases were also searched for in the area of immunological markers, markers of inflammation and microbiota (6) in order to complete the catalogue of indications for participation in screening. Because of the limited sensitivity of radiographic screening, the aforementioned research projects examine other options ranging from PCR-based tests to chromatograms.

Studies on behavioural interventions verified the effectiveness of involving patients in the decision-making process and the effectiveness of community medicine interventions in relation to quitting smoking and participating in LDCT screening (7). It was verified whether the way of providing information about screening using graphics is more effective and associated with more positive attitudes towards screening than the way of providing information based only on text (8). The effectiveness of interventions using video material in motivating people to participate in screening was also verified (9). The effectiveness of behavioural interventions, including motivational interviewing and involving patients in the decision-making process regarding participation in screening is under analysis, as well (10). The effectiveness is examined in relation to the decision to participate in screening and in relation to the time in which patients present for screening. Also, the effectiveness of this type of intervention carried out specifically in rural environments, where fewer patients participated in screening than in cities, was analysed (11). Potential barriers to quitting smoking and participating in screening were the subject of interviews with personnel in a qualitative study (12). In the aforementioned research projects, the necessary conditions for the effectiveness of behavioural interventions and their limitations are addressed. The main remedy, which is under validation, is the empowerment of participants by involving them in the decision-making process. This measure is also under examination in the context of rural environments where the access to medical services is limited.

A study was also carried out on the effectiveness of the use of a virtual doctor who, through telephone calls and text messages, supported patients already participating in screening in sticking to their decision to quit smoking (13). A virtual tool to which women after mammography have access and which is aimed at providing information about indications and increasing the awareness of lung cancer screening is also being evaluated (14). Educational interventions regarding the issue of lung cancer and participation in screening addressed to all adults from groups whose economic and social status hinders access to medical services were also analysed (15). One of the currently conducted studies concerns the evaluation of a multimodal preventive programme. The programme involves taking acetylsalicylic acid to reduce chronic inflammation and supporting quitting smoking by taking cytisine, making changes in dietary behaviour and engaging in physical activity, as well as LDCT screening (16). Virtual tools and educational interventions are under examination. Combining multiple measures in the effort of enhancing the effectiveness of behavioural interventions seems to be the main direction.

## 4. Discussion

Taking into account the high position of lung cancer on the list of causes of death among oncological diseases worldwide, it should be stated that the number of reported studies conducted in the area of screening and the early diagnosis of this type of cancer is disappointingly small. Research on diagnostic tests included an evaluation of the procedure of supplementing LDCT with other diagnostic methods that were performed in parallel to the main diagnostic test or preceded tomography. The effectiveness of behavioural interventions focused on providing information and involving patients in the decision-making process to quit smoking and/or undergo LDCT was also analysed in situations where this procedure was recommended due to the fulfilled diagnostic criteria.

An example of existing solutions in the area of diagnostics is the Lung Cancer Test Program conducted in Poland from 2021 as part of the National Oncology Strategy [16]. The programme is implemented by 31 medical entities and involves LDCT in patients at a high risk of lung cancer. Risk groups are defined based on criteria regarding age, smoking, including periods of abstinence, and other risk factors such as an exposure to chemical risks due to the profession performed, previous cancer diseases of patients and/or their family members and the occurrence of chronic lung diseases such as chronic obstructive pulmonary disease or idiopathic pulmonary fibrosis. Conducting LDCT as part of this programme is aimed at increasing the percentage of detected lung cancer that would be detected in the early stages, increasing the 5-year survival rate and reducing mortality as well as the costs of lung cancer treatment on a national scale.

In the context of behavioural interventions, it is worth noting the 5A algorithms published by the World Health Organization [17] (Ask, Advice, Assess, Assist, Arrange), which can be used in primary health care for patients who smoke tobacco and intend to quit smoking, and for people who are exposed to passive smoking, as well as the 5R algorithm (Relevance, Risks, Rewards, Roadblocks, Repetition) aimed at motivating people to quit smoking. These algorithms determine how medical workers in primary care can conduct conversations with patients. According to the recommendations [18], the question about smoking should be asked during a clinical interview at least once a year. The H2Q (help to quit) method takes into account the level of patients’ motivation to quit smoking and adjusts the treatment accordingly. Ways to deal with addiction are discussed with motivated patients. The results of the literature review and meta-analyses [19] indicate that the high effectiveness of non-pharmacological methods was recorded in 51.72% of studies and the probable effectiveness in 31.38% of studies. Non-pharmacological interventions have not yet been incorporated into routine care in primary care. Doctors and other medical professionals indicate a lack of time as the main reason for the lack of implementation of non-pharmacological interventions supporting coping with tobacco addiction.

The Cochrane network, based on the results of 75 studies, states that the use of pharmacological nicotine replacement therapy is effective in 18 to 25 cases per 100 people addicted to nicotine [20]. Partial nicotinic receptor agonists allow to maintain moderate levels of dopamine by counteracting withdrawal symptoms (acting as an agonist) and also through reducing the satisfaction from smoking (acting as an antagonist). Varenicline is indicated as the most effective pharmacological agent, being more effective than the bupropion and cytisine included in one of the studies discussed (16). However, one review of 75 studies [21], covering a total of 45,049 patients, also confirmed the effectiveness of cytisine, which turned out to be more effective than the placebo without causing any serious neuropsychiatric or cardiac side effects. The same review of studies concluded that cardiac side effects are more likely to occur with varenicline.

In the face of the persistently high mortality rate due to lung cancer, conducting research on complementing the already-developed diagnostic procedures and research on behavioural interventions aimed at promoting the use of tests and quitting smoking seems to be an optimal solution. However, smoking is not the only cause. At least two studies found that lung cancer is diagnosed in women who have never smoked more often than in men [22,23]. 

Unfortunately, the number of these studies seems disproportional in relation to the scale of the problem. The results of the query did not include, for example, research on e-cigarettes. E-cigarettes are sometimes used as a type of replacement therapy for patients addicted to smoking. However, the literature does not clearly indicate the effectiveness of this method [24]. E-cigarettes are also studied as a risk factor in the context of lung cancer because they contain substances shown to be carcinogenic in studies directly related to e-cigarettes, such as nicotine derivatives, heavy metals and flavourings [25].

Also, the query did not include satisfactory number studies regarding specific mutations of critical driver genes, identifying key mutational events and providing methodological frameworks for future findings [26]. Among the recently published papers, some examine the prospects of cyclodextrin-based supramolecular nanomedicine [27], conditions triggering chiroptical inversion behaviours in nanomedicine [28], advancements in supramolecular chemotherapy based on host–guest interactions [29], the use of nucleic acid biosensors in imaging-guided therapy [30] and a combination of macrocyclic chemistry with co-crystal engineering [31,32].

## 5. Conclusions

Throughout recent years, there has been a development of screening procedures and treatment in lung cancer. However, smoking still remains one of the main risk factors and the most significant modifiable factor in lung cancer development. In the face of the persistently high mortality rate, conducting research on complementing the already-developed diagnostic procedures and research on behavioural interventions aimed at promoting the use of tests and quitting smoking is recommended.

## Figures and Tables

**Table 1:** Distribution of clinical trial analysis results in the ClinicalTrial.Gov registry according to the adopted categories.

		Time Frame: 2019–31 March 2024					
No.	Group	Title	Years	*n*	Status	Trial	Population
(1)	Diagnostic	A Multicentre Clinical Trial of Sputum DNA Testing for Lung Cancer in China	2022–2023	659	completed	observational	b.o.
(2)	tests	AI for Lung Cancer Risk Definition in Computed Tomography Screening Programs	2023-	650	ongoing	observational	smokers, aged 50–75
(3)		Feasibility of Cell-Free DNA Liquid Biopsy in Screening High-Risk Patients for Lung Cancer	2022-	100	ongoing	interventional	smokers, aged 50–80
(4)		Evaluation of a Scalable Decision Support and Shared Decision Making Tool for Lung Cancer Screening	2020-	12,000	ongoing	interventional	people meeting the inclusion criteria for LDCT screening
(5)		Breathomics: May it Become an Affordable, New Tool for Early Diagnosis and Screening of Lung Cancer?	2021–2023	60	completed	observational	adults, clinical group and control group
(6)		Structuring of a Lung Cancer Screening Program Including Clinical, Radiological and Biological Phenotyping Useful for the Development of Individualized Risk Prediction Tools: PREVALUNG ETOILE	2023-	160	ongoing	interventional	aged 45–75, lung diseases, meeting inclusion criteria for lung cancer screening
(7)	Behavioural	Inpatient Smokers and LDCT Screening Part 2	2019–2020	128	completed	interventional	people meeting the inclusion criteria for LDCT screening
(8)	interventions	The Impact of Picture Narrative Format on Print Lung Screening Communication Outcomes	2021	326	completed	interventional	people aged 49–75
(9)		Health Opportunities and Promoters of Equitable Screening for Lung Cancer	2023-	2000	ongoing	observational	smokers, aged 50–80
(10)		Proactive Outreach and Shared Decision Making in Improving Lung Cancer Screening Rates in Primary Care Patients	2019-	2355	ongoing	interventional	smokers, aged 55–80
(11)		Shared Decision Making in Rural Primary Care Lung Cancer Screening and Smoking Cessation	2020–2023	118	completed	interventional	smokers, aged 50–80
(12)		Designing an Implementation Strategy for Lung Screening and Smoking Cessation Treatment in Community Health Centres	2022–2023	9	completed	interventional	personnel working with at-risk patients
(13)		CONNECT: Smoking Cessation and Lung Cancer Screening	2019–2023	99	completed	interventional	smokers scheduled for LDCT, aged 55–80
(14)		A Study to Develop a Strategy to Increase Lung Cancer Screening in Women Who May Be at Risk for Lung Cancer	2021-	150	ongoing	interventional	women meeting the inclusion criteria for LDCT screening who had no lesions on mammography
(15)		Awareness, Information, and Resources for Lung Cancer Screening Program for Community-Partnered Lung Cancer Screening	2020–2022	292	completed	interventional	adults
(16)		Screening and Multiple Intervention on Lung Epidemics	2019-	2000	ongoing	interventional	smokers, aged 55–75

## Data Availability

No new data were created during preparation of this manuscript.
